# Osteogenic Potential of Dental Mesenchymal Stem Cells in Preclinical Studies: A Systematic Review Using Modified ARRIVE and CONSORT Guidelines

**DOI:** 10.1155/2015/378368

**Published:** 2015-05-28

**Authors:** Murali Ramamoorthi, Mohammed Bakkar, Jack Jordan, Simon D. Tran

**Affiliations:** ^1^Craniofacial Tissue Engineering and Stem Cells Laboratory, Faculty of Dentistry, McGill University, Montreal, QC, Canada; ^2^King Fahad Armed Forces Hospital, Jeddah, Saudi Arabia

## Abstract

*Background and Objective*. Dental stem cell-based tissue engineered constructs are emerging as a promising alternative to autologous bone transfer for treating bone defects. The purpose of this review is to systematically assess the preclinical in vivo and in vitro studies which have evaluated the efficacy of dental stem cells on bone regeneration. *Methods*. A literature search was conducted in Ovid Medline, Embase, PubMed, and Web of Science up to October 2014. Implantation of dental stem cells in animal models for evaluating bone regeneration and/or in vitro studies demonstrating osteogenic potential of dental stem cells were included. The preferred reporting items for systematic reviews and meta-analyses (PRISMA) guidelines were used to ensure the quality of the search. Modified ARRIVE (Animal research: reporting in invivo experiments) and CONSORT (Consolidated reporting of trials) were used to critically analyze the selected studies. *Results*. From 1914 citations, 207 full-text articles were screened and 137 studies were included in this review. Because of the heterogeneity observed in the studies selected, meta-analysis was not possible. *Conclusion*. Both in vivo and in vitro studies indicate the potential use of dental stem cells in bone regeneration. However well-designed randomized animal trials are needed before moving into clinical trials.

## 1. Introduction

Bone is a multifunctional organ that provides protection, structure, and mechanical support to the body [1]. The integrity of human bone is challenged by infections, trauma, congenital malformation, and surgical removal of tumor [2–4]. Repair and regeneration of bone are a series of biological events involving a number of cell types and signaling pathways in a temporal and spatial sequence [2–6]. When these natural mechanisms/events are compromised, bone grafting is commonly used to augment bone repair and regeneration. Autologous bone grafting has been considered as a “gold standard” because it possesses osteogenesis (osteoprogenitor cells), osteoinduction (BMPs, growth factors), and osteoconduction (scaffold) [7]. However, limitations such as a limited supply, resorption, donor site morbidity, deformity, chronic infection, and rejection demand other alternative treatment approaches [7, 8].

Cell-based bone tissue engineering emerges as a potential alternative as it aims to generate new cell-driven, functional tissue rather than to fill a defect with a nonliving scaffold. It is a combination of principles of orthopedic surgery with biology, physics, material science, and engineering [7]. Classic bone tissue engineering is comprised of osteogenic cells (to form bone tissue matrix), morphogenic signals (help the cells to be the desired phenotype), biocompatible scaffold (to mimic an extracellular matrix niche), and vascular supply (to meet the nutrient supply and clearance of the growing tissue) [7, 8]. Stem cells play a pivotal role in bone tissue engineering [9–15].

Multipotent mesenchymal stromal cells (commonly referred to as mesenchymal stem cells, MSCs) are the most frequently used cell population in tissue engineering because of its multilineage potential, multiple sources, and ability to self-renew [16, 17]. Bone marrow-derived mesenchymal stem cells (BMMSCs) are being considered as a gold standard [7, 9, 16, 17]. However, because of the difficulty to harvest a sufficient cell number as well as the pain and morbidity involved during the harvesting procedure, researchers have been exploring other sources/locations for MSCs. Many anatomical locations have been researched to yield MSC populations [1, 7, 18, 19]. One of the potential sources identified was the dental/oral tissues. Research on using MSCs of dental origin has increased exponentially in the last decade [20–22].

Dental stem/progenitor cells were isolated, characterized, and categorized into six major types [22, 23]: (1) dental pulp-derived stem cells/postnatal dental pulp stem cells (DPSCs), (2) stem cell from exfoliated human dentition (SHED), (3) stem cell from the apical papilla (SCAP), (4) periodontal ligament-derived stem cells (PDLSCs), (5) dental follicle-derived stem cells (DFSCs), and (6) gingival mesenchymal stem cells (GMSCs). The major attractions towards using dental MSCs are ease of access, less invasive approach for harvest, ability to produce higher colony forming units (CFUs), and a higher cell proliferation rate and survival time than bone marrow-derived MSCs [24, 25].

A significant body of literature has been published in the past five years on various types of dental MSCs and its applications [24]. However, there is still limited evidence regarding the capacity of dental MSCs for bone regeneration. An in-depth review and understanding of preclinical in vitro and in vivo studies is a prerequisite to assess the efficacy of dental MSCs and to translate their use into the clinics [26]. Thus the aim of this paper is to perform a systematic review of the literature on dental MSCs for bone regeneration, including in vitro and in vivo studies.

## 2. Materials and Methods

### 2.1. Review Protocol

We focused our review question to address: “Do dental-derived stem cells possess osteogenic potential and regenerate bone defects in in vitro and in animal models”?

### 2.2. Search Strategy

A comprehensive literature search published up to September 2014 was performed on the article databases: Ovid Medline, Embase, PubMed, and Web of Science. The search strategy used a combination of medical subject headings (MeSH) terms and keywords for Medline, PubMed, Web of Science, and EMBASE. The keywords and MeSH terms used for the search were stem cells, mesenchymal stromal cells, progenitor cells, tooth, dental pulp, dental sac, periodontal ligament, deciduous tooth, neural crest, gingiva, SCAP, DPSC, DFSC, GMSC, PDLSC, SHED, bone repair, bone regeneration, bone transplantation, bone substitute, bone tissue engineering, tissue engineering, bone reconstruction, bone defect, osteogenesis, tissue scaffolds, bioreactor, bone morphogenetic protein, intercellular signaling peptide, in vitro, in vivo, animal model, and preclinical. In addition, a hand search strategy was performed by the authors from the citation/reference list of the primary studies and reviews.

### 2.3. Outcomes Measure

- Osteogenic potential/calcified nodule formation/mineralized tissue formation with evidence of osteocyte/osteoblast confirmed by either histology or alkaline phosphatase (ALP) assay or histochemical staining for in vitro studies.
- New bone formation/bone regeneration/defect closure/defect bridging/hard tissue formation (bone)/mineralized tissue or calcified tissue (evidence of osteoblast/osteocyte) confirmed at least by histology or radiography for in vivo studies.

### 2.4. Inclusion Criteria

The selection was limited to the studies which should have

- used at least one type of stem cell derived from dental tissue,
- studied either osteogenic potential or bone regeneration,
- evaluated at least one of the outcomes mentioned above.

### 2.5. Exclusion Criteria

Studies those used Mesenchymal stem cells derived from mandibular bone, maxillary bone, palatal bone, alveolar bone, buccal mucosa. Conference proceedings, abstracts, expert opinion, and letters were excluded from the initial search phase. The manual examination of titles and abstracts further excluded studies that did not meet the inclusion criteria. Odontogenic/periodontal ligament/cementum/dentin regeneration systematic reviews, clinical studies, and non-English articles were omitted after the proofreading of full-text articles.

### 2.6. Screening Methods and Data Extraction

The studies were selected and screened by two authors (Murali Ramamoorthi and Mohammed Bakkar). Disagreements between the reviewers were resolved by consensus with all the authors. Data were extracted based on authors, year of publication, population characteristics (animal species, gender, age, weight, number of animals, stem cell source, intervention, defect location and dynamics, scaffold/carrier/cues, period of observation, and evaluation methods) for in vivo studies, experimental characteristics (stem cell source, osteogenic medium, scaffold/carrier/cues, and evaluation methods) for in vitro studies, and methodological characteristics (study quality/risk bias assessment) for both in vivo and in vitro studies.

### 2.7. Study Quality Assessment

As there are no established sets of criteria/guidelines for assessing the quality or risk of bias for in vivo and in vitro studies [27–32], we assessed the quality of all selected full-text articles using the ARRIVE (animal research: reporting in in vivo experiments) guidelines [27] for in vivo and a modified ARRIVE combined with CONSORT (consolidated reporting of trials) guidelines for in vitro experiments, based on the previous studies [25, 26, 28–30]. The evaluation was based on a predefined grading system of the checklist for in vitro studies (Table 1) and (Table 2) for in vivo studies.

The quality of the articles was assessed by the authors using a checklist of ARRIVE (animal research: reporting in in vivo experiments) guidelines for in vivo studies and using modified ARRIVE and CONSORT (consolidated reporting of trials) guidelines for in vitro studies (the evaluation was based on predefined grading system) (Table 2).

Risk of bias is commonly used to assess clinical trials. Thus we included a risk of bias assessment, as suggested by Bright et al. [25] and the Cochrane Review handbook to improve the quality of our review on dental MSCs. The parameters used were (i) power calculation to determine the samples, (ii) allocation concealment, randomization/replication/multiple experiments done to show consistency, and (iii) blinding in allotment/evaluation of results. A simple Yes or No was used to score selected articles, based on these parameters above.

### 2.8. Statistical Analysis

Because of heterogeneity of sources of dental MSCs, different animal species, diverse defect characteristics, various evaluation times, and different scaffolds/cues among our selected 137 articles, a (statistical) meta-analysis for quantitative review was not possible. We were able to perform a qualitative systematic review.

## 3. Results

### 3.1. Search Results

**Figure 1:**
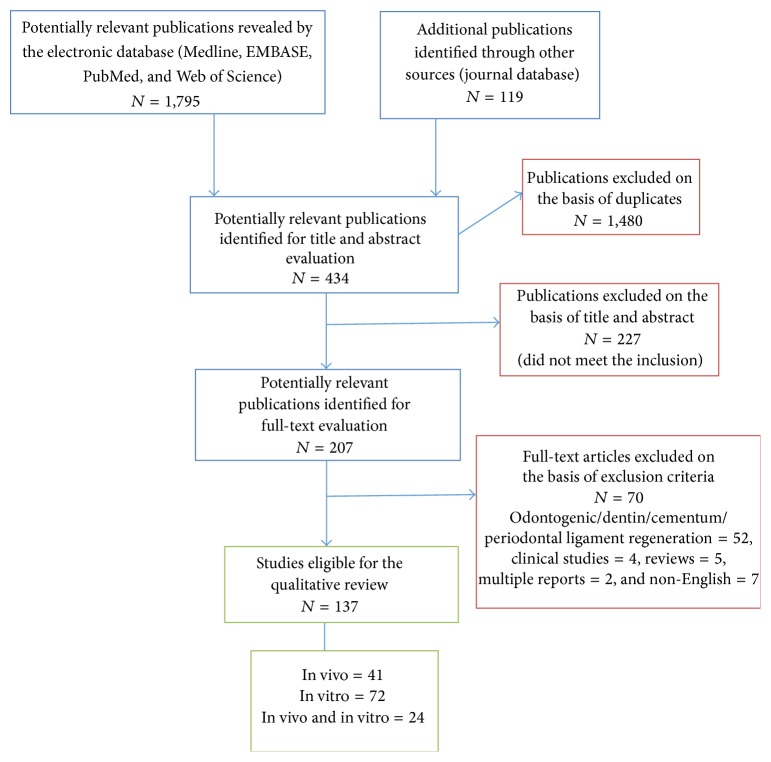
Flow chart demonstrating the strategy used to identify in vitro and in vivo studies for this systematic review of dental stem cells on bone regeneration (PRISMA guidelines is used to design this search strategy).

A total of 1,914 articles were retrieved from the literature search; 1,480 were excluded because of duplication. Four hundred and thirty-four articles were eligible for title and abstract screening. 227 articles were excluded as they did not meet the inclusion criteria. Thus 207 articles were qualified for full-text evaluation. 70 articles were excluded after proofreading the full text. The reasons for exclusion were as follows: odontogenic/dentin/cementum/periodontal ligament regeneration (*n* = 52), clinical studies (*n* = 4), reviews (*n* = 5), language restrictions (*n* = 7), and multiple reports of the same experiment (*n* = 2), thus leaving 137 full articles to be included in this systematic qualitative review. The outline of articles selection is summarized in a flow chart (Figure 1). The details of the included studies are described in Table 3.

### 3.2. Characteristics of the Selected Studies

Out of 137 articles, 80.5% of the studies were published between 2010 and September 2014. Dental pulp-derived (35.5%) and periodontal ligament-derived (30.4%) stem cells were more predominantly studied among the eight different dental sources of stem cells reported in this review. Detailed characteristics (year, source, species, scaffolds/cues, medium, transplanted cell number, evaluation methods, and conclusion of the study) of these studies are shown in Tables 4 and 5.

Five different species of animals (rat/mice, dog, minipig, rabbit, and sheep) were used for the in vivo experiments. A total of 704 animals were used to study the osteogenic potential/bone regeneration of dental stem cells. Out of 65 in vivo studies, 46 used either rats or mice, 13 used dogs, two used minipigs, three used rabbits, and one used sheep to transplant dental stem cells. In 39 out of 65 studies, the dental stem cell source was from humans. Then 13 studies used dental MSCs from dogs, seven from a rat source, two from rabbits, two from minipigs, one from porcine, and one from sheep. The defect type and location were not uniform. Twenty-four studies used subcutaneous implantation on animals, 12 in periodontal defects, nine in mandibular defects, seven in critical-size defects of the calvarium, three in the renal capsule, and one in maxillary sinus augmentation as a defect model to observe osteogenic potential or bone formation in vivo.

In the selected in vitro studies, 85 of the 96 studies used dental MSCs from humans. The remaining 11 studies obtain dental stem cells from rats (7), porcine (1), dog (1), chimpanzee (1), and macaque nemestrima (1). Four in vitro studies used a bioreactor in their experiments. Ninety studies used osteogenic induction medium with serum, while four studies used serum-free medium and two studies used human serum. Nine in vitro studies and five in vivo studies compared the osteogenic potential of different dental derived stem cells. Most of the studies compared the osteogenic potential of PDLSC and GMSC (3 in vivo, 3 in vitro). All these six studies confirmed that PDLSC showed better osteogenic potential compared to GMSC. Based on the included studies that compared osteogenic potential of multiple dental stem cells, PDLSC showed better osteogenic differentiation, followed by DPSC and SHED.

Almost all of the selected studies employed histology (in vivo) or ALP assay and histochemical staining (in vitro) to evaluate the outcomes. Among the 65 in vivo studies, only six studies reported no in vivo bone formation seen with dental stem cells (DFCS-2, DPSC-3, and PDLSC-1). The comparisons of in vivo osteogenic differentiation of different dental stem cells are shown in Table 6. The total number of studies in each type of dental stem cell in this comparison is increased due to the five in vivo studies compared to the osteogenic behavior of different dental stem cells.

### 3.3. Quality Assessment of the Selected Literature

In general, most of the studies included some information related to the animals they used. However the majority of the literature lacked the quality based on ARRIVE guidelines. Only two studies reported a sample size calculation, four studies reported blinding in assessment of the outcomes, and 17/65 studies mentioned randomization in their articles. None of the sixty-five studies mentioned the 3Rs (replacement, reduction, and refinement) in their articles. However, one study mentioned that they followed the ARRIVE guidelines.

In 96 in vitro studies, only one study mentioned the power calculation to sample size. Blinding in evaluation was reported in one in vitro study. Sixteen selected in vitro studies gave information that they repeated their experiments or measurement more than once. Supplemental Tables i, ii, iii, and iv (in Supplementary Material available online at http://dx.doi.org/10.1155/2015/378368) summarize the quality of the in vitro and in vivo studies selected in this review.

## 4. Discussion

The purpose of this review was to summarize the role of dental-derived stem cells (dental MSCs) and their effects on the osteogenic differentiation potential and bone regeneration. Both in vivo and in vitro studies were included in this review. In total, 137 studies were qualitatively reviewed. No randomized controlled trials (RCTs) were found in in vivo studies. The in vitro studies were mainly experimental studies on the osteogenic differentiation or factors enhancing/decreasing the osteogenic potential of various dental stem cells. Dental MSCs used in these studies were derived from the dental pulp, apical papilla, dental papilla, gingiva, dental follicle, dental-neural crest, and periodontal ligament.

The literature stated that dental pulp stem cells were the first to be identified as having mesenchymal properties in the year 2000 by Gronthos and coworkers [33]. To date, four clinical studies were reported using dental stem cells for bone regeneration [9, 22, 24]. Due to the paucity of published clinical studies, we did not include clinical studies in this review. We strongly believe that an in-depth appraisal of the literature on preclinical in vivo and in vitro studies is a prerequisite to understanding the efficacy of a new therapeutic approach before its translation into human use. Dental stem cells such as DPSC, SHED, PDLSC, SCAP, and DFSC fulfill the requirements for mesenchymal stem cell as described by the International Society for cellular therapy [34], that is, adhering to plastic, multilineage differentiation potential, positive to stromal cell markers (CD73, CD90, CD105, STRO1, Nanog) and absence of hematopoietic markers (CD14, CD34, CD45).

### 4.1. SCAPs

The soft tissue covering the root apex of developing teeth serves as a source for SCAPs. All the studies reported in humans are a source for obtaining SCAPs for their experiments. The four in vivo studies conducted in rats and mice revealed ectopic bone-like tissue formation seen at 12 weeks. The in vitro study by Wang and colleagues [35] found an interesting observation, that insulin growth factor 1 (IGF-1) enhanced the osteogenic differentiation but weakened the odontogenic differentiation of SCAPs. Studies by Wu and coworkers [36] confirmed that basic-fibroblast growth factor b FGF inhibited the osteogenic differentiation of SCAP.

### 4.2. DFSCs

Among the four in vivo studies conducted in rats/mice, two studies [37, 38] reported a lack of new bone formation by using DFSCs. However the in vitro study conducted by Tsuchiya et al. reported an osteogenic potential with DFSCs in an appropriate osteogenic induction medium. The two failed studies used porcine or rat as their stem cell source [37, 38]. The study done by Honda et al. [39] demonstrated bone formation similar to intramembranous ossification in rat critical sized calvarial defects. In vitro studies showed that BMP-9 and BMP-6 promoted osteogenesis of DFSCs. A later report [40] mentioned that 37°C to 40°C was optimal for osteogenesis and DFSCs lost its osteogenesis at 41°C.

### 4.3. GMSCs

Two different sources were used in the studies (human, dog). Rats/mice and dogs were used to study the bone regeneration effect. All studies showed that GMSCs were capable of undergoing osteogenic differentiation and forming new bone in the defect area. The cell number used to transplant ranged from 1 × 10^6^ to 5 × 10^6^.

### 4.4. SHEDs

Being a biological waste, SHEDs are an interesting candidate for stem cell therapies. Studies showed that they were capable of rapid proliferation and more frequent population doubling than bone marrow-derived MSCs. In vitro studies confirmed the osteogenic differentiation that rigid scaffolds supported osteogenesis, and bovine fibroblast growth factor inhibited osteogenesis. Almost all the in vivo studies used scaffolds; HA/TCP was the most frequently used carrier. All the in vivo studies confirmed the osteogenic differentiation and bone regeneration potential of SHEDs. A recent report showed that 5-year cryopreserved SHEDs were able to proliferate and undergo osteogenesis without immune reaction in a 9 mm mandibular defect in dogs [41].

### 4.5. DPSCs

Stem cell derived from dental pulp was the most studied dental stem cell for bone regeneration. Among the twenty in vivo studies, three reported that DPSCs were not able to regenerate new bone in subcutaneously implanted mice. Two studies by Annibali et al. in 2013 and 2014 [42, 43] failed to show new bone formation using human DPSCs. Zhang et al. in 2008 [44] demonstrated no evidence of bone formation in mice with rat DPSCs. Almost all the studies used scaffold. Laino et al. in 2005 [45] was able to generate in vitro living autologous bone (LAB) tissue from DPSCs, on subcutaneous implantation in rats LAB remodeled to lamellar bone in 4 weeks.

### 4.6. PDLSCs

PDLSC studies showed diverse source in obtaining periodontal ligament cell. More than half of the in vivo studies used dogs as a source to obtain PDLSCs, and the periodontal defect model was widely used to assess the osteogenic potential. Seo et al. [46] showed human PDLSCs failed to generate new bone in rat periodontal defects after 8 weeks of observation. Ibandronate, simvastin, VEGF, LMHF, BMP 2, and BMP 6 all seemed to enhance osteogenic potential of PDLSCs [47–50]. Silvério et al. [51] in 2010 demonstrated deciduous derived PDLSCs promoted more mineral nodule formation compared to PDLSC derived from permanent teeth in vitro.

Studies by Yamada et al. [52] showed PDLSCs derived from dog and puppy sources were able to generate 10 mm diameter mandibular defects with high vascularity. Wang et al. [53] demonstrated SHEDs have more osteogenic potential than DPSC in mice. Studies confirmed that PDLSC had more osteogenic and bone formation potential than GMSCs [54, 55]. However, Yang et al. [56] studies showed GMSCs had better osteogenic potential than PDLSCs in inflammatory conditions. On average, the 3rd cell passage was used in most of the studies and the addition of scaffolds or growth factors (except b-FGF) improved osteogenesis of the dental stem cells. Although some studies used critical sized defect, most of these studies used either a small size defect or subcutaneous implantation. This jeopardized the extrapolation on outcomes in clinical situations.

Among the various osteogenic induction and growth factors (BMP, IGF, dexamethasone, VEGF, EGF, and FGF) used in the selected studies, it lacks information about the cost effectiveness, safety, and clinical relevance information. Future research should aim to address these parameters.

Most of the selected studies used FBS for culturing dental stem cells. Serum supplementation is important in ex vivo expansion of these cells for clinical use. Using serum containing medium during stem cell culture for human cell therapy is unsafe as it may transfer viral/prion disease, xenogenic antibodies especially if repeated infusions are needed [57]. While FBS based medium may be acceptable for preclinical studies, xeno-free medium is required for expanding these cells in large scale good manufacturing practices (GMP) for clinical applications [57–59]. Furthermore human cells have the possibility to take up animal proteins and present them on their membranes; thus initiating xenogeneic immune response leads to rejection [58]. As the serum condition can significantly affect cell response, it is important to obtain research data with more clinical relevance [58, 59]. Future studies are recommended to compare the safety and efficacy, surface antigen expression, stemness, growth potential, osteogenic differentiation potential of different dental stem cells cultured in FBS, serum-free medium, allogenic human serum, autologous human serum, plasma rich protein, and plasma lysate.

To increase the scientific validity of animal studies, experiments should be appropriately designed, analyzed, and reported transparently. This not only maximizes scientific knowledge, but also is for ethical and economic reasons [30]. The robustness of the research increases by using sufficient animals to achieve scientific objectives and using appropriate statistical analyses to maximize the validity of the experimental outcomes [31]. Using the NC3Rs (National Center for replacement, refinement and reduction of animals in research) ARRIVE guidelines, we performed a detailed analysis of the quality of reporting and statistical analysis of the included in vivo studies. The analysis revealed a number of issues relating to reporting omissions. The majority of the articles reported age of the animals used. However, there was a lack of information about the weight, gender, and housing conditions of the animals used. The availability of online supplementary results offered by many journals to include additional information results negates the argument that researchers are constrained by the page limit [26, 31]. In some of the in vivo studies (*n* = 18/65), the number of animals were simply not reported anywhere in the methodology, results, or discussion sections. Reporting the number of animals is essential to replicate the experiments or to reanalyze the data. Furthermore, 63 of 65 studies did not mention how the sample size was chosen. Determining sample size by power size or simple calculations help to design an animal research with an appropriate number of animals to detect a biologically important effect [28–32]. We cannot rule out that the researchers may have calculated/determined the number of animals but did not report that in the article. However, reporting omission can be easily rectified, as incomplete reporting means potentially flawed research [28].

In vitro preclinical research is the basic foundation for any new therapeutic approach. Although it may not replicate a dynamic environment, in vitro research provides valuable information for future research steps. The methodological quality analysis of the selected in vitro articles revealed the possibility of selection bias. Most of the articles lacked randomization, blinding, sample size calculation, and repetition of the experiments. This affects the scientific validity of experimental results. Although CONSORT guidelines are designed to be used in RCTs, we found it reasonable to apply these guidelines to in vitro studies to emphasize the quality and importance of avoiding bias in reporting or in research, because all phases of research process are interlinked [26, 28, 32]. An inadequate sample size might report incorrect results, which could eventually result in failed animal studies or clinical trials. Comparing the performance of dental stem cells with autologous bone grafts or adipose-derived MSCs or BMMSCs will be an interesting approach. Immune modulation property shown by most of the dental stem cells may provide a solution for graft rejection.

To date few clinical cases of bone tissue engineering used dental stem cells [9, 22, 24]. The main reason for the slow progress is attributed to the extrapolation of outcome from preclinical studies. Based on our observation with the selected literatures and guidelines [26–32, 60], we believe that animal study design should include well defined inclusion and exclusion criteria (study setting), a period to test the participating animals short term ability to adhere to the experimental/treatment regimen (run in period), process of random allocation of animals to the different study groups (randomization), reporting of baseline characteristics (age, sex, and weight) for the all animals in the experimental and control group, animal housing conditions, blinding in outcome assessment and data analyses, clear reporting of number of animals enrolled, followed up, and any addition or number of animals dropped out (attrition), disclosing any adverse effects to the animals during and after intervention/experiment, reporting sample size and methods used to do sample size calculation, and reporting confidence interval in addition to *P* value (for the effect estimate and precision). These parameters will minimize the risk of confounding and selection bias. It also ensures that the outcome of the study is not affected by conscious or unconscious bias or factors unrelated to biological action. Thus improving the internal and external validity of the study. Further well designed and conducted animal randomized control trials (RCTs) will help us to generate high level of scientific evidence similar to human RCTs.

In summary, although selected studies showed dental stem cells have remarkable potential for use in bone regeneration, further well designed preclinical studies addressing optimal differentiating factors, culture medium, critical sized defect model, comparison of osteogenic potential of different dental progenitor cells, biological activity, cost effectiveness, efficacy, and safety of dental stem cells are required before clinical translation.

## 5. Conclusion

Several dental tissues identified by this review possessed dental MSCs with an osteogenic differentiation in vitro and in vivo. Regenerating lost bone tissue was feasible with dental MSCs. The easy accessibility to obtain dental MSCs made them an attractive alternative to BMMSCs for use in clinical trials to evaluate their safety and efficacy. However the current limitation, based on the quality of the literature, requires better designed in vitro or randomized control animal trials before going into clinical trials.

## Supplementary Material

The details of the quality score and risk of bias assessment grading achieved by the final included studies were briefed in the supplementary material. Supplementary Table i , ii, shows the details of the quality score achieved by the selected invitro & invivo studies in each domain [ the characteristics of each domain for invitro and invivo were described in Table 1, 2 in main text]and Tables iii & iv shows the risk of the bias assessment grading for the selected studies.

## Figures and Tables

**Table 1:** Categories used to assess the quality of selected in vitro studies (modified from the ARRIVE and CONSORT guidelines) [26].

Item	Description	Grade
1	Title	(0) Inaccurate/nonconcise (1) Concise/adequate

2	Abstract: either a structured summary of background, research objectives, key experiment methods, principal findings, and conclusion of the study or self-contained (should contain enough information to enable a good understanding of the rationale for the approach)	(1) Clearly inadequate (2) Possibly accurate (3) Clearly accurate

3	Introduction: background, experimental approach, and explanation of rationale/hypothesis	(1) Insufficient (2) Possibly sufficient/some information (3) Clearly meets/sufficient

4	Introduction: preprimary and secondary objectives for the experiments (specific primary/secondary objectives)	(1) Not clearly stated (2) Clearly stated

5	Methods: study design explained number of experimental and control groups, steps to reduce bias (demonstrating the consistency of the experiment (done more than once), sufficient detail for replication, blinding in evaluation, etc.)	(1) Clearly insufficient (2) Possibly sufficient (3) Clearly sufficient

6	Methods: precise details of experimental procedure (i.e., how, when, where, and why)	(1) Clearly insufficient (2) Possibly sufficient (3) Clearly sufficient

7	Methods: How sample size was determined (details of control and experimental group) and sample size calculation.	(1) No (2) Unclear/not complete (3) Adequate/clear

8	Methods: Details of statistical methods and analysis (statistical methods used to compare groups)	(1) No (2) Unclear/not complete (3) Adequate/clear

9	Results: explanation for any excluded data, results of each analysis with a measure of precision as standard deviation or standard error or confidence interval	(1) No (2) Unclear/not complete (3) Adequate/clear

10	Discussion: interpretation/scientific implication, limitations, and generalizability/translation	(0) Clearly inadequate (1) Possibly accurate (2) Clearly accurate

11	Statement of potential conflicts and funding disclosure	(0) No (1) Yes

12	Publication in a peer-review journal	(0) No (1) Yes

**Table 2:** Categories used to assess the quality of selected in vivo studies (based on the ARRIVE guidelines).

Item	Description	Grade
1	Title	(0) Inaccurate/nonconcise (1) Concise/adequate

2	Abstract: either a structured summary of background, research objectives, key experiment methods, principal findings, and conclusion of the study or enough information to enable good understanding of the rationale for the approach (self-contained)	(1) Clearly inadequate (2) Possibly accurate (3) Clearly accurate

3	Introduction: background, experimental approach, and rationale	(0) Insufficient (1) Possibly sufficient/some information (2) Clearly meets/sufficient

4	Introduction: primary and secondary objectives	(0) Not clearly stated (1) Clearly stated

5	Methods: ethical statement (nature of the review permission, relevant license, and national guidelines for the care and use of animals)	(1) Clearly insufficient (2) Possibly sufficient (3) Clearly sufficient

6	Methods: study design explained number of experimental and control groups, steps to reduce bias by allocation concealment, randomization, and binding	(1) Clearly insufficient (2) Possibly sufficient (3) Clearly sufficient

7	Methods: precise details of experimental procedure (i.e., how, when, where, and why)	(0) Clearly insufficient (1) Possibly sufficient (2) Clearly sufficient

8	Methods: experimental animal species, strains, sex, development stage, weight, and source of animals	(1) Clearly insufficient (2) Possibly sufficient (3) Clearly sufficient

9	Methods: housing and husbandry conditions (welfare related assessments and interventions include type of cage, bedding material, number of cage companions, temperature, light or dark cycle, and access to food and water)	(1) Clearly insufficient (2) Possibly sufficient (3) Clearly sufficient

10	Methods: total number of animals used in each experimental group and sample size calculation	(1) No (2) Unclear/not complete (3) Adequate/clear

11	Methods: allocation animals to experimental groups (randomization or matching), order in which animals were treated and assessed	(1) No (2) Yes

12	Methods: outcomes (clearly defines the experimental methods to evaluate the prespecified outcomes)	(1) No (2) Unclear/not complete (3) Clear/complete

13	Methods: details of statistical methods and analysis	(0) No (1) Unclear/not complete (2) Adequate/clear

14	Results: baseline data (characteristic and health status of animals)	(0) No (1) Yes

15	Results: numbers analyzed and explanation for any excluded	(0) No (1) Unclear/not complete (2) Adequate/clear

16	Results for each analysis with a measure of precision as standard error or confidence interval	(1) No (2) Unclear/not complete (3) Yes

17	Adverse events details and modification for reduction	(0) No (1) Unclear/not complete (2) Yes

18	Discussion: interpretation/scientific implication, limitations including animal model, implication for the 3 Rs (replacement, reduction, and refinement)	(1) Clearly inadequate (2) Possibly accurate (3) Clearly accurate

19	Discussion: generalizability/translation	(0) Clearly inadequate (1) Possibly adequate (2) Clearly adequate

20	Statement of potential conflicts and funding disclosure	(0) No (1) Unclear/not complete (2) Yes

**Table 3:** The details and number of studies included in this qualitative review.

Dental stem cell source	In vivo	In vitro	Both in vivo and in vitro
Dental papilla	0	1	0
Apical papilla	0	4	4
Dental follicle	1	6	3
Neural crest	0	1	0
Gingiva	2	0	1
Dental pulp of exfoliated deciduous teeth	5	5	2
Dental pulp of deciduous/permanent teeth	14	29	6
Periodontal ligament	16	19	6
Multiple dental source	3	7	2

**Table:** (a) Stem cells from apical papilla (SCAPs)

Reference	Cell source	Species	Gender	Age Week/months	Weight (mg/kg)	Total number of animals	Defect type and location	Transplanted cell number	Scaffold/growth factors/cues	Period	Evaluation methods	Observation
Abe et al. 2008 [61]	Human	Rat	na	na	na	na	SC pouch	5 × 10^5^	HA	12 wk	Histology	Ectopic bone like tissue on the border of the scaffold

Abe et al. 2012 [62]	Human	Mice	M	4 wk	na	na	SC pouch	5 × 10^4^	Porous HA	12 wk	Histology	Ectopic bone like tissue on the border of the scaffold

Wang et al. 2013 [63]	Human	Mice	na	na	na	12	Renal capsule	1 × 10^6^	Absorbable gelatin sponge	2 wk	Histology	Calcified tissue formation

Qu et al. 2014 [64]	Human	Mice	F	10 wk	na	na	SC	4 × 10^6^	HA/TCP BMP4	8 wk	Histology	DLX2 overexpression enhances mineralized tissue formation.

**Table:** (b) Dental follicular stem cells (DFCSs)

Reference	Cell source	Species	Gender	Age Week/months	Weight (mg/kg)	Total number of animals	Defect type and location	Transplanted cell number	Scaffold/growth factors/cues	Period	Evaluation methods	Observation
Xu et al. 2009 [37]	Rat	Mice	na	na	na	na	Sc pouch	4 × 10^6^	3D-*β* TCP BMP 2	8 wk	Histology	Lacked new bone formation

Tsuchiya et al. 2010 [38]	Porcine	Rat	na	na	na	12	CSD calvarium 5 mm	1 × 10^6^	None	1 wk 4 wk	Histology	No new bone formation. Apparent bone like structure

Honda et al. 2011 [39]	Human	Rat	na	na	na	24	CSD calvarium 8 mm	2 × 10^6^/pellet	None	1 wk 4 wk	Histology	Bone formation with evidence of vascular invasion similar to intramembranous ossification

Park et al. 2012 [65]	Human	Mice	m	8 wk	na	4	SC pouch	1 × 10^6^	DBM Fibrin glue	4 wk	CT Histology	Trabecular bone generation with vessels

**Table:** (c) Gingival mesenchymal stem cells (GMSCs)

Reference	Cell source	Species	Gender	Age Week/months	Weight (mg/kg)	Total number of animals	Defect type and location	Transplanted cell number	Scaffold/growth factors/cues	Period	Evaluation methods	Observation
Wang et al. 2011 [66]	Human	Rat Mice	F	6–8 wk 8–10 wk	160–180 g na	10 3	Mandibular body defect (5 × 2 × 1 mm) SC pouch	na 5 × 10^6^	Type 1 collagen	Histology	8 wk 6 wk	Bone formation in the defected area

Yu et al. 2014 [67]	Dog	Dog	M	na	10-11 kg	4	Class III furcation defect		eGFP	Histology	8 wk	Enhanced new bone formation GMSC (47.11 ± 7.91%) versus control group ( 37 ± 9.53)

Xu et al. 2014 [68]	Human	Mice	M	7 wk	na	36	Rt mandibular body (1.5 mm diameter)	1 × 10^6^	GFP as marker	Histology	1 wk 2 wk 3 wk	Active bone formation at 3 wk

**Table:** (d) Stem cells from human exfoliated dentition (SHEDs)

Reference	Cell source	Species	Gender	Age Week/months	Weight (mg/kg)	Total number of animals	Defect type and location	Transplanted cell number	Scaffold/growth factors/cues	Period	Evaluation methods	Observation
Miura et al. 2003 [69]	Human	Mice	na	na	na	na	SC	2 × 10^6^	HA/TCP	8 wk	Histology	Induce new bone formation

Seo et al. 2008 [70]	Human	Mice	na	na	na	18	Calvaria (2.7 mm)	2 × 10^6^	HA/TCP	6–8 wk 6 month	Histology	Robust bone formation without hematopoietic bone marrow

Zheng et al. 2009 [71]	Minipig	Minipig	F	4–6 m	20–30 kg	16	Bilateral parasymphyseal CSD (2.5 × 1.5 × 1.5 cm^3^) *N* = 10 1 × 1 × 0.5 cm^3^ *N* = 6	2 × 10^7^ to 4 × 10^8^	PT67/eGFP *β* TCP HA/TCP	24 wk 2 wk [3] 4 wk [3]	*µ*-CT Histology	Defects restored with new bone at 6 m

Li et al. 2012 [72]	Human	Mice	F	8–12 wk	na	na	SC pouch	4 × 10^6^	HA/TCP bFGF	8 wk	Histology	b FGF downregulated STRO-1, CD146, CD90, and CD73 expression of SHED

Vakhrushev et al. 2012 [73]	Human	Mice	na	na	na	na	na	na	3D PLGA	1 month	DAPI staining	More intense expression of osteocalcin on scaffolds with SHED

Alkaisi et al. 2013 [74]	Human	New Zealand Rabbit	na	3–5 months	2.7 ± 0.31 kg	22	Distraction of 6.2 mm between first lower premolar and mental foramen	6 × 10^6^	None	2 wk 4 wk 6 wk	Radiology Histology	New bone formation with thick cortices and marrow cavity at 6 wk

Behina et al. 2014 [41]	Human SHED 5 yr ago	Dog	M	na	15–25 kg	4	Mandibular through-through (9 mm diameter)	na	Collagen	12 wk	Histology	5-year cryopreserved SHED able to proliferate and osteogenesis without immune response. Bone formation is same as control group

**Table:** (e) Dental pulp derived stem cells (DPSCSs) from deciduous/permanent teeth

Reference	Cell source	Species	Gender	Age Week/months	Weight (mg/kg)	Total number of animals	Defect type and location	Transplanted cell number	Scaffold/growth factors/cues	Period	Evaluation methods	Observation
Laino et al. 2006 [75]	Human (deciduous teeth)	Rat	na	10–12 wk	na	5	SC	Woven bone obtained by in vitro SHED culture	Woven bone	4 wk	Histology	Woven bone remodeled to lamellar bone with osteocytes entrapped within the lamella

Otaki et al. 2007 [76]	Human	Mice	na	7 wk	na	na	SC	2 × 10^6^ to 1.8 × 10^7^	HA/TCP	7 wk 15 wk	Histology	50% bone formation seen

de Mendonça Costa et al. 2008 [77]	Human	Rat	M	4 months	320–420 gm	8	Cranium (5 × 8 mm)	1 × 10^6^	Collagen membrane	7 d 20 d 30 d 60 d 120 d	Histology	Defect healed with new bone formation

Zhang et al. 2008 [44]	Rat	Mice	na	10 wk	na	10	SC	5 × 10^6^	HA/TCP	5 wk 10 wk	Histology	No evidence of bone formation

Morito et al. 2009 [78]	Human	Mice	na	10 wk	na	na	SC	4 × 10^5^	PLGA with Calcium Phosphate	5 wk 10 wk	Histology	Confirmed bone and cartilage formation

Yang et al. 2009 [79]	Rat	Mice	na	10 wk	na	12	SC	5 × 10^6^	AdBMP-2 HA/TCP	1 wk 4 wk 12 wk	Histology	Enhance mineral tissue formation

Kraft et al. 2010 [80]	Human	Mice	F	8 wk	na	2	1.5 cm deep pouch	5 × 10^5^	HA-TCP	8 wk	Histology	Lamellar bone like structure

Chan et al. 2011 [81]	Human	Mice	na	6 wk	na	5	SC pouch	1 × 10^5^	SAPN	4 wk	Histology	Mineralized tissue formed

Ito et al. 2011 [82]	Dog	Dog	na	2 yr	na	3	Hemimandible 10 × 10 mm	1 × 10^7^	PRP gel	8 wk	Histology	Significant amount of new bone formation seen in the defect

Li et al. 2011 [83]	Human	Mice	na	6 wk	na	8	SC	na	None	4 wk	Histology X ray	Bone formation seen.

Liu et al. 2011 [84]	Rabbit	New Zealand Rabbit	F	Na	2.5–3 kg	36	Segmental 10 × 4 × 3 mm	1 × 10^8^	n HAC/PLA rh-BMP-2 eFG	12 wk	Histology X ray	Bone regenerated in the defect area

Pisciotta et al. 2012 [85]	Human	Rat	M	14 wk	na	10	5.8 × 1.5 mm cranial	1 × 10^6^	Collagen sponge	6 wk	Histology	Regeneration of resected bone

Riccio et al. 2012 [86]	Human	Rat	M	12–14 wk	na	15	5 × 8 mm parietal	na	Silk fibroin	4 wk	Histology	Induce new bone formation in the critical sized defect

Annibali et al. 2013 [42]	Human	Mice	na	50 days	na	75	Parietal (4 × 1 mm)	1 × 10^6^	DBB *β* TCP Hydrogel-ceramic composite sponge	1 wk 2 wk 4 wk 8 wk	Histology	TE constructs did not significantly improve bone regeneration

Khorsand et al. 2013 [87]	Dog	Dog	M	1-2 yr	14–22 kg	10	3 × 5 × 8 mm	2 × 10^7^	BIO-OSS	8 wk	Histology	Woven bone formation seen and no significant difference seen between control and experimental group

Maraldi et al. 2013 [88]	Human	Rat	M	12–14 wk	na	30	Parietal 5 × 8 mm	na	Collagen	4 wk 8 wk	Histology	New bone formation seen in the defect

Wang et al. 2013 [89]	Rat	Rat	F	8 wk	na	30	Ovariectomy Renal capsule	1 × 10^6^	Absorbable gelatin sponge	14 days	Histology	Estrogen deficiency inhibits osteogenic potential of DPSCS (downregulated by NF-κB pathway)

Annibali et al. 2014 [43]	Human	Rat	na	50 days	na	8	Parietal (5 × 1 mm)	na	GDPB *β* TCP	2 wk 4 wk 8 wk 12 wk	*µ*-CT *µ*-PET	Addition of stem cell did not increase new bone formation

Ling et al. 2014 [90]	Rabbit	New Zealand Rabbit	na	na	2.5–3 kg	6	SC	1 × 10^6^	n HAC/PLA *β* TCP	8 wk	Histology	Mature bone formation seen

Niu et al. 2014 [91]	Human	Mice	M	5 wk	na	6	SC	5 × 10^6^	ISCS NCS	8 wk	Histology	New bone formation seen.

**Table:** (f) Periodontal ligament derived stem cells (PDLSCs)

Reference	Cell source	Species	Gender	Age Week/months	Weight (mg/kg)	Total no of animals	Defect type and location	Transplanted cell number	Scaffold/growth factors/cues	Period	Evaluation methods	Observation
Doğan et al. 2002 [92]	Dog	Dog	na	na	na	1	Class II furcation defect	2 × 10^5^	Blood clot	42 days	Histology	PDLSC promote bone regeneration

Seo et al. 2004 [46]	Human	Rat Mice	na	12–10 wk	na	Rat-6 Mice-12	Rat-2 mm^2^ periodontal defect Mice-SC	Rat-2 × 10^6^ Mice-4 × 10^6^	HA-TCP	6–8 wk	Histology	No bone formation seen

Murano et al. 2006 [93]	Dog	Dog	na	na	na	15	Class III furcation defect	na	None	2 wk 4 wk 8 wk	Histology	Bone regeneration with filling of most defect along with cementum formation

Iwata et al. 2009 [94]	Dog	Dog	M	na	10 kg	4	3-wall defect (5 × 5 × 4 mm)	na	PGA	6 wk	Histology Micro-CT	Significant new bone formation compared to control group

Kim et al. 2009 [95]	Dog	Dog	M	na	12–15 Kg	4	Mandibular 5 × 10 mm saddle defect	1 × 10^6^	HA/TCP	16 wk	Histology	Defect regenerated new bone

Ding et al. 2010 [96]	Minipig	Minipig	M & F	6–8 m	30–40 kg	15	3 × 7 × 5 mm periodontal defect	2-cell sheet/defect	HA/TCP	0 wk 12 wk	CT-Scan Histology	PDLSC sheet repair allogeneic bone defect

He et al. 2011 [97]	Dog	Dog	na	2 year	na	na	SC pocket	2 × 10^6^	nHAC/PLA	8 wk	Histology	New bone like tissue seen

Grimm et al. 2011 [98]	Human	Rats	na	10 wk	na	17	2.5 × 2.5 × 2 mm^3^ periodontal defect	1 × 10^5^	Collagen sponge	2 wk 6 wk 8 wk	Histology	PDLSC able to regenerate bone

Lee et al. 2012 [47]	Human	Mice	M	6–8 wk	na	na	SC	na	HA/TCP VEGF FGF-2	8 wk	Histology	Hard tissue formation seen.

Suaid et al. 2012 [99]	Dog	Dog	na	1.46 ± 0.18 years	10–20 kg	7	Bilateral Class III defect	3 × 10^5^	Collagen	12 wk	Histology	New bone formation seen in the defect

Tour et al. 2012 [100]	Rat	Rat	M	na	350 gm	24	CSD Calvaria 8 mm	2 × 10^5^	HA-ECM	12 wk	Histology	Bone regeneration observed in the CSD

Yu et al. 2012 [48]	Human	Mice	na	na	na	na	Renal capsule	1 × 10^6^	Absorbable gelatin sponge IGF-1	6–8 wk	Histology	IGF-1 enhances osteogenic differentiation of PDLSC Immature bone like structure formed

Gao et al. 2013 [101]	Human	Mice	M	4–6 wk	na	12	SC	na	Osthole HA-TCP	4 wk	Histology	Significant bone formation seen

Ge et al. 2013 [102]	Human	Rat	M	8 wk	180–220 gm	18	Bilateral parietal defect 5 mm diameter	1 × 10^7^	HGCCS GCF	12 wk	Histology	Bone formation seen in the defect

Mrozik et al. 2013 [103]	Sheep	Sheep	na	3–5 years	63.5–72 kg	13	Rectangular 0-wall defect (10 mm deep)	1 × 10^7^	Gelfoam	4 wk	Histology	New alveolar bone formation seen, not significant with gelfoam alone group but significant with control group

Yu et al. 2013 [104]	Rat	Rat	na	7 wk	na	12	Bilateral 3 wall bone defect (2 × 2 × 1.7 mm^3^)	4 × 10^6^	Gelatin sponges	6 wk	Histology	New bone formed in the defect

Han et al. 2014 [105]	Rat	Rat	F	na	220–250 g	36	Periodontal defect	1 × 10^6^	Gel foam	1 wk 2 wk 3 wk 4 wk	Histology	Complete bridging of osseous defect with mineralized tissue containing osteocytes

Jung et al. 2014 [106]	Human	Mice	na	6 wk	na	14	SC	na	rAD-EGFP hBMP2	2 wk 8 wk	Histology	Ectopic Bone formation seen

Park et al. 2015 [107]	Dog	Dog	na	na	10–12 kg	6	Peri-implantitis	na	HA Ad BMP2	7.5 months	Histology	New bone formation and re osseointegration of implants seen

Yu et al. 2014 [108]	Dog	Rat	M	2 m	150 g	24	CSD calvaria (4 mm wide)	2 × 10^6^	Bio-oss	8 wk	Micro-CT Histology	Defect regenerated new bone

Yu et al. 2014 [109]	Dog	Dog	M	18 m	14.5 kg	6	Maxillary sinus floor augmentation	2 × 10^6^	Bio-oss	8 wk	Micro-CT Histology	New bone formation seen

Zhao and Liu 2014 [110]	Human	Mice	na	na	na	na	SC	4 × 10^6^	Ceramic bovine bone simvastatin	8 wk	Histology	Bone like hard tissue formation on the scaffold. Larger amount seen in PDLSC and scaffold with simvastatin group

**Table:** (g) Multiple dental stem cells

Reference	Cell source	Type compared	Species	Gender	Age Week/months	Weight (mg/kg)	Total number of animals	Defect type and location	Transplanted cell number	Scaffold/growth factors/cues	Period	Evaluation methods	Observation
Yamada et al. 2011 [52]	Dog	c DPSC p DTSC	Dog	na	2 yr	na	na	Three 10 mm diameter mandibular defects	na	PRP	8 wk 16 wk	Histology	Well-formed new bone with vascularity is seen in all groups studied.

Wang et al. 2012 [53]	Human	SHED DPSC	Mice	na	8 wk	na	na	SC	2 × 10^6^	CBB Fibrin gel	8 wk	Histology	Higher osteogenic differentiation and bone formation seen in SHED compared to DPSC.

Moshaverinia et al. 2013 [54]	Human	PDLSC GMSC	Mice	na	5 months	na	na	SC	2 × 10^6^	Injectable alginate hydrogel	8 wk	Micro-CT Histology	ALP activity as well as mineralized tissue formation of PDLSC is better than GMSC but comparatively less than BMMSC.

Yang et al. 2013 [56]	Human	PDLSC GMSC	Mice	M	6 wk	na	na	SC	2 × 10^5^	Artificial bone repair material	8 wk	Histology	Significant bone formation seen. However GMSC demonstrated better osteogenic potential and bone formation in inflammatory condition compared to PDLSC.

Moshaverinia et al. 2014 [55]	Human	PDLSC GMSC	Mice	na	5 months	na	16	5 mm diameter calvarial defect	4 × 10^6^	RGD-coupled alginate	8 wk	Micro-CT Histology	Bone regeneration in defect area (greater in BMMSC, moderate in PDLSC, lesser in GMSC groups)

**Table:** (a) Stem cells from apical papilla (SCAPs)

Reference	Cell source	Medium	Scaffold/carriers/cues/markers	Evaluation methods	Observation
Abe et al. 2008 [61]	Human	OIM	HA	ALP assay Staining, SEM	Time dependent ALP activity seen.

Park et al. 2009 [111]	Human	OIM	None	Histochemical staining	Osteoblast differentiation and mineralized nodule formation seen.

Abe et al. 2012 [62]	Human	OIM	None	Histochemical staining	SCAPs differentiate into osteoblasts, adipocytes, chondrocytes, and smooth muscle.

Wang et al. 2012 [35]	Human	OIM	IGF-1	ALP assay Histochemical staining	IGF-1 enhances osteogenic differentiation but weakens odontogenic differentiation of SCAPs.

Wu et al. 2012 [36]	Human	OIM	bFGF	ALP assay Histochemical staining	SCAP cultured with bFGF shows decreased mineralized nodule formation and ALP activity, but if pretreated with bFGF increased mineralized nodule formation is seen.

Wang et al. 2013 [63]	Human	OIM	None	ALP assay Histochemical staining	High ALP activity and RUNX2 upregulation seen.

Qu et al. 2014 [64]	Human	OIM	None	ALP assay Histochemical staining	Significant mineralization observed and enhanced osteogenesis is linked to DLX2.

**Table:** (b) Dental papilla stem cells

Reference	Cell source	Medium	Scaffold/carriers/cues/markers	Evaluation methods	Observation
Ikeda et al. 2006 [112]	Human	OIM	HA	ALP assay	In vitro osteogenic differentiation observed if cultured in presence of OIM.

**Table:** (c) Dental follicular stem cells (DFCSs)

Reference	Cell source	Medium	Scaffold/carriers/cues/markers	Evaluation methods	Observation
Tsuchiya et al. 2010 [38]	Porcine	OIM	None	ALP assay Histochemical staining	DFCS has osteogenic potential.

Honda et al. 2011 [39]	Human	GCM	None	ALP assay Histochemical staining	3 distinct cell populations were identified with DFCS. Among the three, two of them showed strong calcium accumulation.

Viale-Bouroncle et al. 2011 [113]	Human	OIM	Polydimethylsiloxane Fibronectin	ALP assay	Soft surface improved the induction of osteogenesis differentiation of DFSC compared to higher stiffness.

Aonuma et al. 2012 [114]	Human	OIM	None	ALP assay Histochemical staining	ALP activity higher than hMSC.

Li et al. 2012 [115]	Rat	OIM	Ad-BMP9 Ad-GFP	Histological staining	BMP 9 enhances osteogenesis of DFCS.

Park et al. 2012 [65]	Human	OIM	None	Histochemical staining	DFSC able to undergo osteogenic differentiation.

Mori et al. 2012 [116]	Human	OIM	None	ALP assay Histochemical staining	High level of ALP expression, osteogenic potential, and mineralized nodule formation seen.

Rezai Rad et al. 2013 [40]	Rat	OIM	None	ALP assay Histochemical staining	Osteogenesis of DFSC increased with temperature from 37°C to 40°C but lost its potential at 41°C.

Takahashi et al. 2013 [117]	Human	OIM	None	ALP assay	DFSC can undergo osteogenic differentiation in the absence of dexamethasone and BMP 6 is the key gene in osteogenic differentiation of DFSC.

Yao et al. 2013 [118]	Rat	OIM	hr-BMP6	ALP assay	DFSC lost its osteogenesis during in vitro expansion; addition of BMP-6 dramatically enhances osteogenesis of late passage.

**Table:** (d) Gingival mesenchymal stem cells (GMSCs)

Reference	Cell source	Medium	Scaffold/carriers/cues/markers	Evaluation methods	Observation
Yu et al. 2014 [67]	Human	OIM	None	ALP assay Histochemical staining	Mineralized nodule formed in the experimental group.

**Table:** (e) Dental neural crest stem cells

Reference	Cell source	Medium	Scaffold/carriers/cues/markers	Evaluation methods	Observation
Degistirici et al. 2010 [119]	Human	OIM	None	ALP assay Histology	Bone like matrix formation seen.

**Table:** (f) Stem cells from human exfoliated dentition (SHEDs)

Reference	Cell source	Medium	Scaffold/carriers/cues/markers	Evaluation methods	Observation
Miura et al. 2003 [69]	Human	OIM	rhBMP 4	Histochemical staining	Osteogenic differentiation observed.

Vakhrushev et al. 2010 [120]	Human	Serum-free OIM	3D polylactide matrix	Histochemical staining	SHED and BMMSC have similar phenotype and identical osteogenic potential.

Li et al. 2012 [72]	Human	OIM	bFGF	Histochemical staining	bFGF inhibits osteogenic induction.

Viale-Bouroncle et al. 2012 [121]	Human	OIM	PDMS Fibronectin	ALP assay Histochemical staining	Rigid scaffold supports proliferation and osteogenesis of SHED.

Vakhrushev et al. 2013 [122]	Human	Serum-free OIM	TCP	Histochemical staining	TCP increases osteogenic differentiation, ossification foci and enhances ECM production by SHED.

Karadzic et al. 2014 [123]	Human	OIM	3D HAP, PLGA, alginate, EVA/EVV	ALP assay Histology	All four are suitable carrier for SHED. Low level of osteoblastic differentiation is demonstrated in EVA/EVV.

Yu et al. 2014 [124]	Human	OIM	None	ALP assay Histochemical staining	ALP activity and in vitro mineralization were not different between SCID and SHED. However more TNF-*α* is seen with SCID.

**Table:** (g) Dental pulp derived stem cells (DPSCSs) from deciduous/permanent teeth

Reference	Cell source	Medium	Scaffold/carriers/cues/markers	Evaluation methods	Observation
Gronthos et al. 2000 [33]	Human	OIM	None	ALP assay	DPSC shows osteogenic potential (formed condensed nodule with high level of calcium) and forms more CFU than BMMSC.

Laino et al. 2005 [45]	Human	OIM	None	ALP assay Histochemical staining	DPSC able to generate living autologous fibrous bone tissue (LAB).

Laino et al. 2006 [75]	Human	OIM	None	Calcium staining	Demonstrated pluripotency. Able to differentiate into osteoblast.

d'Aquino et al. 2007 [125]	Human	OIM	None	ALP assay Histochemical staining	DPSC able to form woven bone in vitro.

Cheng et al. 2008 [126]	Chimpanzee	OIM	None	Histochemical staining	Osteogenic capacity of cDPSC was comparable to human BMMSC, DPSC, and rBMSC.

Graziano et al. 2008 [127]	Human	OIM Rotating culture	HA, Ti, PLGA	ALP assay Histochemical staining	PLGA shows better scaffold suitability for DPSC (1 mm bone tissue on PLGA, 0.3 mm in Ti, and no bone tissue formation seen in titanium covered with HA).

Morito et al. 2009 [78]	Human	OIM	PLGA bFGF	ALP assay Histochemical staining	Membrane bone like tissue formed around PLGA.

Alge et al. 2010 [128]	Rat	OIM	None	ALP assay Histochemical staining	Significantly higher ALP activity than control group.

Han et al. 2010 [129]	Human	OIM Mechanical bioreactor	None	ALP assay Histochemical staining	Mechanical stimulation promotes osteogenic differentiation and osteogenesis of DPSC.

Mangano et al. 2010 [130]	Human	OIM	LST Ti	Histology SEM	More osteoblast and bone formation seen with laser treated titanium surface.

Mori et al. 2010 [131]	Human	OIM	None	ALP assay	DPSC formed mineralized matrix nodules showing osteoblast features.

Spath et al. 2010 [132]	Human	OIM	Lenti virus vector expressing *β* galactoside	ALP assay Histochemical staining	DPSC by explant culture method exhibits elevated proliferation and osteogenic potential.

Chan et al. 2011 [81]	Human	OIM	SAPN	Histochemical staining	DPSC survives encapsulation by SAPN and calcium salt deposition seen.

Galli et al. 2011 [133]	Human	OIM	3DTi	ALP assay Histochemical staining	Increased expression of ALP genes and BMP 2 genes and increased osteogenic differentiation.

D'Alimonte et al. 2011 [134]	Human	OIM	VEGF-A165 peptide	ALP assay Histochemical staining	VEGF enhances differentiation of DPSC towards osteoblast and DPSC showed negative hematopoietic marker.

Li et al. 2011 [83]	Human	OIM	3D gelatin	ALP assay Histochemical staining	Increased ALP activity and osteoblast compared to control group.

Mangano et al. 2011 [135]	Human	OIM	Biocoral	Histology SEM	Diffuse bone formation seen in the scaffold.

Struys et al. 2011 [136]	Human	OIM	None	TEM Staining Image analysis	Presence of multiple mineralization nuclei.

Huang et al. 2012 [137]	Rat	OIM	Flavanoid	ALP assay Histochemical staining	Flavonoid increases DPSCs ALP activity by 1.47-fold and upregulation of RUNX2by 2.5-fold.

Huang et al. 2012 [138]	Rat	OIM	MAO Ti	ALP assay	Osteogenic potential of DPSC similar to BMMSC.

Khann-Jain et al. 2012 [139]	Human	Human serum (serum-free OIM)	*β*TCP	ALP assay Histochemical staining	Matrix mineralization seen. Human serum can be substituted for FBS which facilitates translating from in vitro to clinical trials.

Pisciotta et al. 2012 [85]	Human	Human serum OIM	Collagen sponge	ALP assay Histochemical staining	High proliferation rate and osteogenic differentiation of DPSC in human serum compared to FCS.

Taşli et al. 2014 [140]	Human	OIM	BMP2,7 Plasmids, GFP	ALP assay Histochemical staining	Transfection of human tooth germ cells with BMP2,7, induced osteogenic, and odontogenic differentiation.

Palumbo et al. 2013 [141]	Human	OIM	3D scaffold matrigel Titanium	SEM Confocal TEM	Human osteoblasts from bone biopsies are appropriate compared to DPSCs.

Zavatti et al. 2013 [142]	Human	Ferutinin OIM	None	Staining	Ferutinin enhances osteoblastic differentiation of DPSC.

Akkouch et al. 2014 [143]	Human	OIM	3D Col/HA/PLCL	Micro-CT ALP assay Histochemical staining	30% increase in bone nodule formation and tissue mineralization seen on surface as well inside the scaffold.

Amir et al. 2014 [144]	Macaque Nemestrima	Chitosan OIM	None	ALP assay Histochemical staining	Chitosan stimulates proliferation and early osteogenic differentiation of DPSC compared to dexamethasone.

Guo et al. 2014 [145]	Human	OIM	Fluorapatite PCL	ALP assay Histochemical staining	Scaffolds provided favorable ECM microenvironment for proliferation and osteogenic differentiation.

Huang et al. 2014 [146]	Human	OIM	Lenti virus Cloned human OCT4, Nanog	ALP assay Histochemical staining	OCT 4 and Nanog act as a major regulator in maintaining mesenchymal properties in DPSC.

Jensen et al. 2014 [147]	Human	OIM	NSP-PCL HT-PCL	ALP assay Histochemical staining	Both scaffolds promote calcium deposition, but HT-PCL supports only cell proliferation and migration.

Ji et al. 2014 [148]	Human	OIM Biomimetic bioreactor	3D agarose gel	ALP assay Histochemical staining	Mechanical loading enhances osteogenesis and bone formation

Kanafi et al. 2014 [149]	Human	OIM	Alginate hydrogel	Calcium quantification assay Staining	DPSC immobilized in alginate hydrogel exhibits enhanced osteogenic potential

Niu et al. 2014 [91]	Human	OIM cocultured with silicic acid	Collagen	ALP assay Histochemical staining	ISCS promotes proliferation, osteogenic differentiation, and mineralization compared with NCS.

Taşli et al. 2013 [150]	Human	OIM	NaB	ALP assay Histochemical staining	NaB significantly increases level of ALP activity and mineralization with higher expression of osteogenic and odontogenic genes.

Woloszyk et al. 2014 [151]	Human	OIM Spinner flask bioreactor	Silk fibroin	Micro-CT Histology ALP assay	DPSCs have the potential to form mineralized matrix when grown on 3D scaffold enhanced by mechanical loading.

**Table:** (h) Periodontal ligament derived stem cells (PDLSCs)

Reference	Cell source	Medium	Scaffold/carriers/cues/markers	Evaluation methods	Observation
Gay et al. 2007 [152]	Human	OIM	None	Histochemical staining	PDLSC has osteogenic differentiation and mineralization potential.

Trubiani et al. 2007 [153]	Human	OIM	Xenogenic Porcine substitute	ALP assay Histochemical staining	Scaffold able to support PDLSC and demonstrated osteogenic potential.

Zhou et al. 2008 [154]	Human	OIM	None	ALP assay Histochemical staining	Time dependent increase in matrix calcification observed with PDLSC.

Orciani et al. 2009 [155]	Human	OIM	None	TEM SEM ALP assay	NO involved in osteogenesis of PDLSC. In vitro osteogenesis of PDLSC resulted in osteoblast like cells with calcium deposits.

He et al. 2011 [97]	Dog	OIM	Porous n HAC/PLA	ALP assay	Osteogenic differentiation seen on the scaffolds.

Silvério et al. 2010 [51]	Human	OIM	None	Histochemical staining	Deciduous periodontal ligament derived cells promoted 100% mineral nodule formation, while permanent showed 60%.

Zhang et al. 2011 [156]	Rats	OIM	None	Histochemical staining	Decreased osteogenic differentiation seen in PDLSC derived from ovariectomised rats.

Zhou et al. 2011 [49]	Human	OIM	Ibandronate	qRT-PCR	Ibandronate promoted osteoblastic differentiation of PDLSC.

Ge et al. 2012 [157]	Human	OIM	IHGCCS	ALP assay Histochemical staining	HGCS showed higher ALP activity.

Lee et al. 2012 [47]	Human	OIM	VEGF2 FGF2	ALP assay Histochemical staining	VEGF has positive effect on osteogenic differentiation. FGF has positive effect on proliferation rate.

Sununliganon and Singhatanadgit 2012 [158]	Human	OIM	None	Staining	PDLSC able to form mineralized mass.

Yu et al. 2012 [48]	Human	OIM	IGF-1	ALP assay Histochemical staining	IGF-1 stimulates osteogenic potential of PDLSC.

Zhang et al. 2012 [50]	Human	OIM LMHF	None	ALP assay Histochemical staining	LMHF promoted osteogenic potential of PDLSC.

Gao et al. 2013 [101]	Human	OIM	None	ALP assay Histochemical staining	PDLSC able to form mineralized nodule.

Ge et al. 2013 [102]	Human	OIM	HAp PADM	ALP assay Histochemical staining	Higher ALP activity and osteogenic differentiation seen in Hap-PADM than pure PADM.

Houshmand et al. 2013 [159]	Human	OIM	EMD	Histochemical staining	EMD has no effect on osteoblastic differentiation of BMMSC or PDLSC.

Kato et al. 2013 [160]	Human	OIM	Synthetic peptide	ALP assay	More number of calcified nodules seen in culture with synthetic peptide.

Kim et al. 2013 [161]	Human	Hesperetin OIM	None	ALP assay	Significant increase in ALP activity.

Kong et al. 2013 [162]	Human	OIM	None	ALP assay	Periodontal disease derived PDLSC displayed impaired osteogenesis compared to healthy PDLSC.

Singhatanadgit and Varodomrujiranon 2013 [163]	Human	OIM spheroid culture	Conical polypropylene tube	Staining	Bone like deposit seen. PDLSC may undergo osteogenic differentiation in an osteogenic scaffold-free 3D spheroidal culture.

Yu et al. 2013 [164]	Human	OIM	None	ALP assay Histochemical staining	Osteogenic differentiation of PDLSC far superior to WJCMSC.

Hakki et al. 2014 [165]	Human	OIM	Type I collagen BMP6	Histochemical staining	BMP application stimulated mineralized nodule formation.

Jung et al. 2014 [106]	Human	OIM	rAd-EGFP, BMP2	Histochemical staining	Mineralized nodule formation seen. BMP 2 effectively promoted osteogenesis.

Tang et al. 2014 [166]	Human	OIM	None	ALP assay Histochemical staining	PDLSCs have osteogenic potential and low immunogenicity.

Ye et al. 2014 [167]	Human	OIM	Ad-BMP9	ALP assay Histochemical staining	BMP 9 promoted matrix mineralization.

**Table:** (i) Multiple dental stem cells

Reference	Cell source	Comparison	Medium	Scaffold/carriers/cues/markers	Evaluation methods	Observation
Koyama et al. 2009 [168]	Human	DPSC SHED	OIM	BMP2	ALP assay Histochemical staining	No difference observed between DPSC and SHED for osteogenic potential.

Chadipiralla et al. 2010 [169]	Human	SHED PDLSC	Serum-free OIM	Retinoic acid ITS	ALP assay Histochemical staining	High proliferation rate seen in PDLSC makes it a better osteogenic cell source. However SHED is more responsive to retinoic acid.

Bakopoulou et al. 2011 [170]	Human	DPSC SCAP	OIM	None	ALP assay Histochemical staining	DPSC and SCAP positive for markers of both osteogenic and odontogenic differentiation.

Lee et al. 2011 [171]	Human	DPSC PDLSC	PRP OIM	None	ALP assay Histochemical staining	PRP induces osteogenic and odontogenic differentiation.

Atari et al. 2012 [172]	Human	DPSC DPMSC	OIM	3D glass scaffold	ALP assay Histochemical staining	DPPSCs have higher expression of bone markers than DPMSC.

Moshaverinia et al. 2012 [173]	Human	PDLSC GMSC	OIM	Alginate hydrogel	SEM XRD Staining	Osteogenic potential is observed higher for BMMSC followed by PDLSC and lowest in GMSC.

Yang et al. 2013 [56]	Human	PDLSC GMSC	OIM	None	ALP assay Histochemical staining	PDLSC showed more effective osteogenic differentiation than GMSC

Davies et al. 2014 [174]	Human	DPSC ADSC BMSC	OIM	None	Micro-CT Histochemical staining SEM	High volume of mineralized matrix seen in DPSC group but diffused layer of low density seen in SEM.

Moshaverinia et al. 2014 [55]	Human	PDLSC GMSC	OIM	RGD coupled alginate microsphere	Western blot Fluorescent image analysis	Osteogenic potential of BMMSC is greater than PDLSC. However PDLSC shows better osteogenic potential than GMSC. Stem cells encapsulated in RGD showed enhanced osteogenesis.

**Table 6:** Invivo comparison of osteogenic potential different Dental stem cells.

Type of dental stem cells	Total no of selected invivo studies	No. of studies failed to show osteogenic potential	% of Studies showed osteogenic potential
SCAP	4	0	100%
DFCS	4	2	50%
GMSC	6	0	100%
DPSC	22	3	86.36%
SHED	8	0	100%
PDLSC	25	1	96%

## Abbreviations

AdBMP2: Adenovirus carrying bone morphogenetic protein
ALP: Alkaline phosphatase
b FGF: Basic fibroblast growth factor
BMMSC: Bone marrow derived mesenchymal stromal cell
BMP: Bone morphogenetic protein
Cap: Calcium phosphate
CBB: Ceramic bovine bone
cDPSC: Dental pulp stem cell derived from chimpanzee
Col: Collagen
CSD: Critical sized defect
CT: Computed tomography
DFSC: Dental follicle stem cell
DLX2: Distal less homeobox 2
DPSC: Dental pulp stem cell
ECM: Extracellular matrix
EMD: Enamel matrix derivative
F: Female
FBS: Fetal bovine serum
FCS: Fetal calf serum
FGF: Fibroblast growth factor
GCF: Genipin chitosan framework
GCM: Growth culture medium
GFP: Green fluorescent protein
GDPB: Granular deproteinized bone
GMSC: Gingiva derived mesenchymal cell
HAP: Hydroxy apatite
HGCCS: Nanohydroxyl apatite coated genipin chitosan conjugated scaffold
IGF-1: Insulin growth factor
ISCS: Intrafibrillar silicified collagen scaffold
ITS: Insulin transferring selenous acid
Kg: Kilogram
LMHF: Low magnitude high frequency
LST: Laser sintered
m: Month
M: Male
MAO: Mono arc oxygen
Na; na: Not available
nHAC: Nanohydroxyl apatite collagen
OIM: Osteogenic induction medium
PCL: Polycaprolactone
PDMS: Polydimethyl siloxane
PET: Positive emission tomography
PLCL: Poly(L-lactide-co-epsilon-caprolactone)
rh: Recombinant
RGD: Arginine-glycine-aspartic acid tripeptide
SAPN: Self-assembling peptide nanofibre hydrogel
SC: Subcutaneous
SCAP: Stem cell from apical papilla
SCID: Stem cell from inflamed pulp
SEM: Scanning electron microscope
SHED: Stem cell from human exfoliated dentition
Ti: Titanium
TCP: Tricalcium phosphate
TNF-*α*: Tumor necrosis factor-alpha
VEGF: Vascular endothelial growth factor
Wk: Week
WJCMSC: Wharton jelly of umbilical cord stem cells.
