# Supplementary material for: Osteogenic Potential of Dental Mesenchymal Stem Cells in Preclinical Studies: A Systematic Review Using Modified ARRIVE and CONSORT Guidelines
Source: Stem Cells Int. 2015 May 28;2015:378368. doi: 10.1155/2015/378368 (PMC4464683; doi:10.1155/2015/378368)
Supplement: Supplementary file 1 — The details of the quality score and risk of bias assessment grading achieved by the final included studies were briefed in the supplementary material. Supplementary Table i , ii, shows the details of the quality score achieved by the selected invitro & invivo studies in each domain [ the characteristics of each domain for invitro and invivo were described in Table 1, 2 in main text]and Tables iii & iv shows the risk of the bias assessment grading for the selected studies. [file 378368.f1.pdf]

Supplemental Table i : Methodological characteristics of In- Vitro Studies based on the guidelines

Studies	1	2	3	4	5	6	7	8	9	10	11	12
**SCAP**												
Abe s et al 2008	1	2	2	1	0	2	0	0	0	1	0	1
Park BW et al 2009	1	2	2	1	0	2	0	0	1	1	0	1
Abe S et al 2012	1	1	2	0	0	2	1	1	1	1	1	1
Wang S et al 2012	1	2	2	1	1	2	0	2	1	1	1	1
Wu J et al 2012	1	2	2	1	1	2	0	2	1	1	1	1
Wang L et al 2013	1	2	2	1	1	2	0	2	1	1	0	1
Qu B et al [2014]	1	1	2	1	1	2	0	2	1	1	1	2
**Dental Papilla**												
Ikeda E et al [2006]	1	0	2	1	0	2	0	1	0	1	0	1
**Dental Follicular**												
Tsuchiya S et al[2010]	1	1	2	1	0	2	1	1	1	2	1	1
Honda MJ et al 2011	1	2	2	1	0	2	1	1	1	1	1	1
Viale Bouroncle S et al[2011]	1	2	2	1	0	2	1	2	2	2	0	1
Aonouma H et al[2012]	1	2	2	1	0	2	1	2	1	2	1	1
Li C et al[2012]	1	1	2	0	0	2	1	2	1	2	1	1
Park BW et al 2012	1	2	2	1	0	2	1	1	1	1	1	1
Mori g et al[2012]	1	2	2	1	0	2	1	2	0	2	1	1
Rezai Rad M et al[2013]	1	2	2	1	1	2	0	1	1	2	1	1
Takahashi K et al[2013]	1	2	2	1	0	2	1	2	1	2	1	1
Yao S et al[2013]	1	2	2	1	1	2	1	1	1	2	1	1
**Gingival**												
Yu X et al[2013]	1	2	2	1	0	1	1	0	0	1	1	1
**Neural Crestal Cells**												
Degistrici o et al[2010]	1	2	2	1	0	2	0	0	1	2	0	1
**SHED**												
Miura M et al 2003	1	2	1	0	0	2	1	1	1	1	1	1
Vakhrusev IV et al[2010]	1	0	0	0	0	2	0	0	0	1	1	1
Li B et al 2012	1	2	1	0	1	1	1	1	1	1	1	1
Viale-Bouroncle S et al[2012]	1	2	2	1	0	2	0	1	1	2	0	1
Vakhrusev IV et al[2013]	1	1	2	0	0	2	0	0	0	1	1	1
Karadzic L et al [2014]	1	1	2	1	0	2	0	2	1	2	0	1
Yu S et al[2014]	1	2	2	1	0	2	1	1	1	2	0	1
**Pulp**												
Gronthos 2000	1	2	2	1	0	2	0	1	0	1	0	1
Laino G et al[2005]	1	1	2	0	1	2	0	0	1	1	1	1
Laino G et al[2006]	1	1	2	0	1	2	0	0	1	1	1	1
D’Aquino R et al [2007]	1	2	2	1	1	2	0	0	1	1	1	1
Cheng PH et al[2008]	1	0	2	1	1	2	0	1	1	1	1	1
Morito A et al 20091	1	1	1	1	1	2	1	1	1	1	1	1
Graziano A et al[2008]	1	1	2	1	0	2	0	1	0	1	1	1
Alge DL et al[2010]	1	2	2	1	1	2	0	2	1	1	1	1
Han MJ et al[2011]	1	2	2	1	1	2	1	2	1	1	1	1
Mangano C et al [2010]	1	2	2	0	1	2	0	2	0	1	1	1
Mori G et al[2010]	1	1	2	1	1	2	0	2	1	1	1	1
Spath I et al[2010]	1	2	2	1	0	2	0	2	0	1	1	1
Chan B et al [2011]	1	2	2	1	0	2	0	2	0	2	0	1
Galli D et al[2011]	1	1	2	0	1	2	0	2	1	1	1	1
Ida et al[2011]	1	1	2	0	1	2	0	2	1	1	1	1
Li JH et al[2011]	1	2	2	1	0	2	0	0	0	1	1	1
Mangano C et al[2011]	1	1	2	1	1	2	0	2	1	1	1	1
Struys T et al[2011]	1	2	2	1	1	2	0	2	0	1	0	1
Huang XF et al[2012]	1	2	2	1	1	2	0	2	1	1	0	1
Huang Y et al[2012]	1	2	2	0	0	2	0	2	1	1	1	1
Khann-Jain R et al[2012]	1	2	2	1	1	2	0	2	1	1	1	1
Pisciotta A et al[2012]	1	2	2	1	1	2	0	2	1	1	1	1
Tasli PN et al[2013]	1	2	2	1	0	2	0	1	1	2	1	1

**Palumbo C et al[2013]**	1	1	2	1	1	2	0	2	1	1	1	1
**Zavatti M et al[2013]**	1	2	2	1	1	2	0	2	2	1	1	1
**Akkouch A et al [2014]**	1	1	2	1	1	2	0	2	1	1	1	1
**Amir LR et al[2014]**	1	2	2	1	0	2	0	2	1	1	0	1
**Guo T et al[2014]**	1	2	2	1	0	2	0	2	1	1	1	1
**Huang CE et al[2014]**	1	2	2	0	1	2	0	2	2	1	1	1
**Jensen J et al[2014]**	1	1	2	1	1	2	0	2	1	1	1	1
**Ji J et al[2014]**	1	2	2	0	0	2	2	2	1	1	1	1
**Kanafi MM et al [2014]**	1	1	2	1	1	2	0	2	1	1	0	1
**Niu LN et al[2014]**	1	1	2	1	0	2	0	2	1	1	1	1
**Tasli PN et al[2014]**	1	2	2	1	0	2	0	1	1	1	0	1
**Woloszyk A et al [2014]**	1	2	2	0	0	2	0	2	1	1	1	1
**PDLSC**												
**Gay IC et al[2007]**	1	2	2	1	1	2	1	2	2	2	1	1
**Trubiani O et al[2007]**	1	2	2	1	0	2	0	0	1	1	1	1
**Zhou Y et al[2008]**	1	2	1	0	1	2	1	2	2	2	1	1
**Orciani M et al[2009]**	1	2	2	1	0	2	0	2	2	2	1	1
**He H et al[2010]**	1	2	2	1	0	2	1	2	1	1	1	1
**Silverio KG et al [2010]**	1	2	2	1	1	2	1	2	2	2	1	1
**Zhang B et al 2011**	1	2	2	1	1	2	1	1	2	1	1	1
**Zhou Q et al[2011]**	1	2	2	1	0	1	0	2	1	1	0	1
**Ge S et al [2012]**	1	2	2	1	0	2	0	2	1	2	1	1
**Lee JH et al [2012]**	1	2	1	0	1	1	1	2	1	1	1	1
**Sunnunliganon L et al[2012]**	1	2	2	1	0	2	0	1	1	1	1	1
**Yu Y et al[2012]**	1	2	2	1	0	1	1	2	1	1	1	1
**Zhang C et al [2012]**	1	2	2	1	1	2	1	2	1	2	1	1
**Gao LN et al [2013]**	1	2	2	1	1	2	1	2	1	2	1	1
**Ge S et al[2013]**	1	1	1	0	0	2	0	2	1	1	1	1
**Houshmand B et al[2013]**	1	2	2	1	1	2	1	2	2	2	1	1
**Kato H et al [2013]**	1	2	2	1	1	2	1	2	2	2	1	1
**Kim SY et al[2013]**	1	2	1	1	0	2	0	2	1	2	1	1
**Kong X et al [2013]**	1	2	1	0	0	2	1	2	1	2	1	1
**Singhatanadgit W et al[2013]**	1	2	2	1	1	2	1	2	2	2	1	1
**Yu S et al[2013]**	1	1	1	0	0	2	1	2	2	2	1	1
**Hakki SS et al[2014]**	1	2	2	1	2	2	1	2	2	2	1	1
**Jung IH et al[2014]**	1	1	2	1	1	2	1	2	1	2	1	1
**Tang R et al[2014]**	1	2	1	0	0	2	0	1	1	1	1	1
**Ye G et al[2014]**	1	1	1	0	1	2	0	2	1	1	1	1
**Multiple Stem Cells**												
**Koyama N et al[2009]**	1	2	1	0	0	1	0	1	1	2	0	1
**Chadipiralla K et al[2010]**	1	2	2	1	1	2	1	2	1	2	1	1
**Bakopoulou A[2011]**	1	2	2	1	1	2	1	2	1	2	1	1
**Lee UL et al[2011]**	1	2	2	1	0	2	1	1	1	2	1	1
**Atari M et al[2012]**	1	2	2	1	1	2	1	2	1	2	1	1
**Moshaverinia A et al[2012]**	1	2	2	1	0	2	1	2	1	2	0	1
**Yang H et al[2010]**	1	2	2	1	1	2	1	2	1	2	1	1
**Davies OG et al [2014]**	1	2	2	1	0	2	1	2	1	2	1	1
**Moshaverina A et al[2014]**	1	2	2	1	0	2	2	2	2	2	1	1

Supplemental Table ii: Methodological characteristics of in vivo Studies based on the guideline

Studies	1	2	3	4	5	6	7	8	9	10	11	12	13	14	15	16	17	18	19	20
**SCAP**																				
**Abe S et al [2008]**	1	1	2	1	1	0	2	0	0	0	0	2	1	0	0	0	0	1	1	0
**Abe s et al[2012]**	1	2	2	1	1	0	2	1	0	0	0	1	0	0	0	1	0	1	1	1
**Wang L et al [2013]**	1	2	2	1	0	0	2	0	0	0	0	0	2	0	0	1	0	1	1	1
**Qu B et al 2014**	1	1	1	1	1	0	1	1	0	0	0	0	1	0	1	1	0	0	0	2
**Dental Follicular**																				
**Xu LL et al[2009]**	1	2	2	1	2	0	1	0	0	0	0	1	1	0	1	1	0	1	1	2
**Tsuchiya S et al[2010]**	1	1	2	1	1	0	1	0	0	0	0	1	1	0	1	1	0	0	2	2
**Honda MJ et al[2011]**	1	2	2	1	2	0	1	0	0	1	0	1	1	0	1	1	1	1	1	2
**Park BW et al [2012]**	1	2	2	1	0	0	2	1	0	0	0	1	2	0	1	1	0	1	1	2
**Gingival**																				

**Wang F et al[2011]**	1	2	2	1	2	1	2	2	2	1	1	2	0	0	1	0	0	1	2	2
**Yu X et al[2013]**	1	2	2	1	1	1	2	1	0	1	1	2	1	0	2	1	1	1	1	2
**Xu QC et al[2014]**	1	1	1	0	2	1	2	1	1	1	1	2	2	0	1	1	0	1	1	2
**SHED**																				
**Miura M et al 2003**	1	2	1	0	0	0	1	0	0	0	0	0	1	0	1	1	1	1	1	2
**Seo BB et al[2008]**	1	2	1	0	1	1	1	1	0	1	0	1	0	0	2	0	0	1	1	0
**Zheng Y et al [2009]**	1	2	2	1	2	0	1	2	2	1	0	1	1	0	1	1	1	0	1	2
**Li B et al[2012]**	1	2	2	1	1	0	1	2	2	1	0	1	1	0	1	1	0	0	1	2
**Vakhrusev IV et al[2012]**	1	1	1	0	0	0	1	0	0	0	0	1	0	0	0	0	0	0	1	2
**Alkaisi A et al[2013]**	1	2	2	1	2	1	2	1	1	1	0	1	2	0	1	1	1	0	1	0
**Behina et al A [2014]**	1	2	2	1	1	2	2	2	2	1	1	2	1	0	2	2	2	1	1	0
**Pulp**																				
**Laino G et al 2006**	1	1	1	0	0	0	1	0	0	1	0	1	1	0	1	1	0	0	1	2
**Otaki S et al[2007]**	1	1	2	0	0	0	2	1	0	0	0	0	0	0	0	1	0	1	1	1
**De Mendonca costa A et al[2008]**	1	2	2	0	2	0	2	1	2	1	0	1	0	0	0	0	2	1	1	2
**Zhang W et al[2008]**	1	1	2	1	2	1	2	1	0	1	1	2	1	1	0	1	2	2	2	0
**Morito A et al [2009]**	1	1	1	1	1	1	1	1	1	1	1	0	1	1	1	2	1	1	1	2
**Yang x ET AL[2009]**	1	2	2	1	2	0	2	1	0	1	0	1	2	1	0	0	1	1	1	1
**Kraft DCE et al[2010]**	1	2	2	1	2	1	2	2	0	0	2	2	0	0	0	2	0	1	1	2
**Chan B et al[2011]**	1	1	2	1	2	0	2	1	0	1	0	1	0	0	0	0	2	1	1	2
**Ito K et al[2011]**	1	1	2	1	2	1	2	0	1	1	1	1	2	0	0	1	0	1	1	2
**Li JH et al[2011]**	1	2	2	1	0	0	2	0	0	0	0	1	0	0	0	0	0	1	1	2
**Liu HCE et al[2011]**	1	1	2	1	2	0	2	1	2	0	0	2	2	0	0	1	2	1	1	0
**Pisciotta A et al[2012]**	1	1	2	1	2	0	2	1	0	1	0	2	2	0	0	1	0	1	1	1
**Riccio M et al[2012]**	1	1	2	1	2	0	2	1	0	1	0	1	1	0	0	1	2	1	1	1
**Annibali S et al 2013**	1	2	2	1	0	0	1	0	1	1	1	1	1	0	1	1	0	0	1	2
**Khorsand A et al[2013]**	1	1	2	1	0	1	2	2	1	0	1	2	2	0	0	1	0	1	1	0
**Maraldi T al [2013]**	1	1	2	1	2	1	2	1	2	0	0	1	2	1	0	1	2	1	1	2
**Wang Y et al [2013]**	1	1	2	0	2	1	2	2	0	1	1	1	2	0	0	1	0	1	1	1
**Annibali S et al[2014]**	1	2	2	1	2	0	2	1	2	1	0	2	2	0	0	2	0	2	2	2
**Ling LE et al[2014]**	1	1	2	1	2	0	2	1	0	1	0	2	0	0	0	2	0	1	1	0
**Niu LN et al [2014]**	1	1	2	1	2	0	2	1	0	0	0	1	2	0	0	0	2	1	1	1
**PDLSC**																				
**Dogan A et al[2002]**	1	2	2	1	2	1	2	0	0	1	1	2	1	0	2	0	1	1	1	0
**Seo BM et al[2004]**	1	2	2	1	2	0	2	1	0	1	1	1	2	0	2	1	1	1	1	2
**Murano et al[2006]**	1	2	2	1	1	0	2	0	0	0	0	1	1	0	1	1	0	1	1	0
**Iwata T et al[2009]**	1	1	1	0	2	0	2	0	0	1	0	1	1	0	1	1	0	1	1	2
**Kim SH et al[2009]**	1	2	2	1	1	1	2	1	0	1	1	1	2	0	2	2	1	1	1	2
**Ding G et al [2010]**	1	2	2	1	2	1	2	2	1	1	1	2	2	0	1	1	1	1	1	2
**He H et al[2010]**	1	2	2	1	1	1	2	0	0	1	1	1	2	0	1	1	1	1	1	2
**Grimm WD et al[2011]**	1	1	1	0	2	1	2	0	0	0	1	1	0	0	0	0	0	1	1	1
**Lee JH et al[2012]**	1	2	1	0	1	0	1	0	1	0	0	1	1	0	1	1	0	1	1	2
**Suaid FF et al [2012]**	1	2	2	1	2	1	2	2	0	1	1	2	2	0	2	2	1	1	1	2
**Tour G et al[2012]**	1	2	2	1	2	1	2	1	1	1	1	2	2	0	2	1	2	1	1	2
**Yu Y et al[2012]**	1	2	2	1	0	0	1	0	0	1	0	1	2	0	1	1	0	0	0	2
**Gao LN et al[2013]**	1	2	2	1	2	1	2	0	0	1	1	2	2	0	2	2	1	1	2	2
**Ge S et al[2013]**	1	2	2	1	0	0	2	1	0	1	0	1	2	0	1	1	0	1	1	2
**Mrozik et al [2013]**	1	2	1	1	2	1	2	2	0	1	1	2	2	0	2	2	1	1	1	2
**Yu N et al[2013]**	1	2	2	1	2	1	2	1	0	1	1	2	2	0	2	1	2	1	1	2
**Han j et al[2014]**	1	2	2	1	2	1	2	1	0	1	1	2	2	0	2	1	2	1	1	2
**Jung IH et al[2014]**	1	1	2	1	2	1	2	2	0	1	0	1	2	0	1	2	1	1	1	2
**Park SY et al[2014]**	1	2	2	0	1	0	1	1	0	1	0	1	1	0	1	1	0	1	1	2
**Yu BH et al[2014]**	1	2	2	1	2	1	2	2	2	1	1	2	2	0	2	2	2	1	2	2
**Yu BH et al[2014]**	1	2	2	1	1	1	2	1	0	1	1	2	2	0	1	1	1	1	1	2
**Zhao BJ [2014]**	1	2	2	1	1	1	2	1	0	1	0	1	1	0	2	1	0	1	1	0
**Multiple**																				
**Yamada Y et al[2011]**	1	1	2	1	2	1	2	1	0	1	0	2	2	0	2	1	2	1	2	2
**Wang X et al[2012]**	1	2	2	1	2	0	2	1	0	1	0	1	2	0	1	1	2	1	2	2
**Moshaverina A et al[2013]**	1	2	2	1	1	1	2	1	1	1	1	1	2	1	2	1	1	1	2	2
**Yang H et al[2013]**	1	2	2	1	2	0	2	0	0	1	1	1	2	0	1	1	0	0	2	2
**Moshaverina A et al [2014]**	1	2	2	1	2	1	2	2	1	2	0	2	2	0	1	1	1	1	1	2

Supplemental Table iii: In - Vitro studies graded with yes or no for risk bias assessment

Studies	Power calculation/Sample size calculation	Allocation concealment	Repetition/ Randomization	Blinding in analysis
**SCAP**				
**Abe s et al 2008**	No	No	No	No
**Park BW et al 2009**	No	No	No	No
**Abe S et al 2012**	No	No	No	No
**Wang S et al 2012**	No	No	Yes	No
**Wu J et al 2012**	No	No	No	No
**Wang L et al 2013**	No	No	No	No
**Qu B et al [2014]**	No	No	No	No
**Dental Papilla**				
**Ikeda E et al [2006]**	No	No	No	No
**Dental Follicle**				
**Tsuchiya S et al[2010]**	No	No	No	No
**Honda MJ et al 2011**	No	No	No	No
**Viale Bouroncle S et al[2011]**	No	No	No	No
**Aonouma H et al[2012]**	No	No	No	No
**Li C et al[2012]**	No	No	No	No
**Park BW et al 2012**	No	No	No	No
**Mori g et al[2012]**	No	No	No	No
**Rezai Rad M et al[2013]**	No	No	No	No
**Takahashi K et al[2013]**	No	No	No	No
**Yao S et al[2013]**	No	No	No	No
**Gingival**				
**Yu X et al[2013]**	No	No	No	No
**Neural crest**				
**Degistrici o et al[2010]**	No	No	No	No
**SHED**				
**Miura M et al 2003**	No	No	No	No
**Vakhrusev IV et al[2010]**	No	No	No	No
**Li B et al 2012**	No	No	No	No
**Viale-Bouroncle S et al[2012]**	No	No	No	No
**Vakhrusev IV et al[2013]**	No	No	No	No
**Karadzic L et al [2014]**	No	No	No	No
**Yu S et al[2014]**	No	No	No	No
**Pulp**				
**Gronthos 2000**	No	No	No	No
**Laino G et al[2005]**	No	No	No	No
**Laino G et al[2006]**	No	No	No	No
**D'Aquino R et al [2007]**	No	No	Yes	No
**Cheng PH et al[2008]**	No	No	No	No
**Graziano A et al[2008]**	No	No	No	No
**Morito A et al 2009**	No	No	Yes	No
**Alge DL et al[2010]**	No	No	No	No
**Han MJ et al[2011]**	No	No	No	No
**Mangano C et al [2010]**	No	No	No	No
**Mori G et al[2010]**	No	No	No	No
**Riccio M et al [2010]**	No	No	No	No
**Spath I et al[2010]**	No	No	No	No
**Chan B et al [2011]**	No	No	No	No
**Galli D et al[2011]**	No	No	No	No
**Ida et al[2011]**	No	No	No	No
**Li JH et al[2011]**	No	No	No	No

**Mangano C et al[2011]**	No	No	No	No
**Struys T et al[2011]**	No	No	No	Yes
**Huang XF et al[2012]**	No	No	No	No
**Huang Y et al[2012]**	No	No	No	No
**Khann-Jain R et al[2012]**	No	No	No	No
**Pisciotta A et al[2012]**	No	No	No	No
**Tasli PN et al[2013]**	No	No	No	No
**Annibali S et al 2013**	No	No	Yes	No
**Palumbo C et al[2013]**	No	No	No	No
**Zavatti M et al[2013]**	No	No	No	No
**Akkouch A et al [2014]**	No	No	No	No
**Amir LR et al[2014]**	No	No	Yes	No
**Guo T et al[2014]**	No	No	No	No
**Huang CE et al[2014]**	No	No	No	No
**Jensen J et al[2014]**	No	No	No	No
**Ji J et al[2014]**	Yes	No	No	No
**Kanafi MM et al [2014]**	No	No	No	No
**Niu LN et al[2014]**	No	No	No	No
**Tasli PN et al[2014]**	No	No	No	No
**Woloszyk A et al [2014]**	No	No	No	No
**PDLSC**				
**Gay IC et al[2007]**	No	No	Yes	No
**Trubiani O et al[2007]**	No	No	No	No
**Zhou Y et al[2008]**	No	No	Yes	No
**Orciani M et al[2009]**	No	No	No	No
**He H et al[2010]**	No	No	No	No
**Silverio KG et al [2010]**	No	No	No	No
**Zhang B et al 2011**	No	No	No	No
**Zhou Q et al[2011]**	No	No	No	No
**Ge S et al [2012]**	No	No	Yes	No
**Lee JH et al [2012]**	No	No	No	No
**Sunnunliganon L et al[2012]**	No	No	No	No
**Yu Y et al[2012]**	No	No	Yes	No
**Zhang C et al [2012]**	No	No	Yes	No
**Gao LN et al [2013]**	No	No	No	No
**Ge S et al[2013]**	No	No	No	No
**Houshmand B et al[2013]**	No	No	No	No
**Kato H et al [2013]**	No	No	No	No
**Kim SY et al[2013]**	No	No	No	No
**Kong X et al [2013]**	No	No	No	No
**Singhatanadgit W et al[2013]**	No	No	No	No
**Yu S et al[2013]**	No	No	No	No
**Hakki SS et al[2014]**	No	No	No	No
**Jung IH et al[2014]**	No	No	No	No
**Tang R et al[2014]**	No	No	No	No
**Ye G et al[2014]**	No	No	No	No
**Multiple Stem Cells**				
**Koyama N et al[2009]**	No	No	Yes	No
**Chadipiralla K et al[2010]**	No	No	Yes	No
**Bakopoulou A[2011]**	No	No	Yes	No
**Lee UL et al[2011]**	No	No	Yes	No
**Atari M et al[2012]**	No	No	Yes	No
**Moshaverinia A et al[2012]**	No	No	No	No
**Yang H et al[2010]**	No	No	No	No
**Davies OG et al [2014]**	No	No	Yes	No
**Moshaverina A et al[2014]**	No	No	Yes	No

Supplemental table –iv: In vivo studies graded with yes or no for risk bias assessment

**Studies**				
**SCAP**	**Power calculation/ Sample size calculation**	**Allocation concealment**	**Repetition/ Randomization**	**Blinding in analysis**
**Abe S et al [2008]**	No	No	No	No
**Abe s et al[2012]**	No	No	No	No
**Wang L et al [2013]**	No	No	No	No
**Qu B et al 2014**	No	No	Yes	No
**Dental Follicular**				
**Xu LL et al[2009]**	No	No	No	No
**Tsuchiya S et al[2010]**	No	No	No	No
**Honda MJ et al[2011]**	No	No	No	No
**Park BW et al [2012]**	No	No	No	No
**Gingival**				
**Wang F et al[2011]**	No	No	Yes	No
**Yu X et al[2013]**	No	No	Yes	No
**Xu QC et al[2014]**	No	No	Yes	No
**SHED**				
**Miura M et al 2003**	No	No	No	No
**Seo BB et al[2008]**	No	No	No	No
**Zheng Y et al [2009]**	No	No	No	No
**Li B et al[2012]**	No	No	No	No
**Vakhrusev IV et al[2012]**	No	No	No	No
**Alkaisi A et al[2013]**	No	No	No	No
**Behina et al A [2014]**	No	No	Yes	No
**Pulp**				
**Laino G et al 2006**	No	No	No	No
**Otaki S et al[2007]**	No	No	No	No
**De Mendonca costa A et al[2008]**	No	No	No	No
**Zhang W et al[2008]**	No	No	No	No
**Morito A et al [2009]**	No	No	No	No
**Yang x ET AL[2009]**	No	No	No	No
**Kraft DCE et al[2010]**	No	No	No	Yes
**Chan B et al[2011]**	No	No	No	No
**Ito K et al[2011]**	No	No	No	No
**Li JH et al[2011]**	No	No	No	No
**Liu HCE et al[2011]**	No	No	No	No
**Pisciotta A et al[2012]**	No	No	No	No
**Riccio M et al[2012]**	No	No	No	No
**Annibali S et al 2013**	No	No	No	No
**Khorsand A et al[2013]**	Yes	No	Yes	No

**Maraldi T al [2013]**	No	No	No	No
**Wang Y et al [2013] Estrogen Study**	No	No	No	No
**Annibali S et al[2014]**	No	No	No	No
**Ling LE et al[2014]**	No	No	No	No
**Niu LN et al [2014]**	No	No	No	No
**PDLSC**				
**Dogan A et al[2002]**	No	No	Yes	No
**Seo BM et al[2004]**	No	No	Yes	No
**Murano et al[2006]**	No	No	No	No
**Iwata T et al[2009]**	No	No	No	No
**Kim SH et al[2009]**	No	No	No	No
**Ding G et al [2010]**	No	No	Yes	No
**He H et al[2010]**	No	No	No	No
**Grimm WD et al[2011]**	No	No	No	No
**Lee JH et al[2012]**	No	No	No	No
**Suaid FF et al [2012]**	No	No	Yes	Yes
**Tour G et al[2012]**	No	No	Yes	No
**Yu Y et al[2012]**	No	No	No	No
**Gao LN et al[2013]**	No	No	No	No
**Ge S et al[2013]**	No	No	Yes	No
**Mrozik et al [2013]**	No	No	Yes	Yes
**Yu N et al[2013]**	No	No	Yes	No
**Han j et al[2014]**	No	No	No	No
**Jung IH et al[2014]**	No	No	Yes	No
**Park SY et al[2014]**	No	No	No	No
**Yu BH et al[2014]**	No	No	Yes	No
**Yu BH et al[2014]**	No	No	Yes	No
**Zhao BJ [2014]**	No	No	No	No
**Multiple**				
**Yamada Y et al[2011]**	No	No	Yes	No
**Wang X et al[2012]**	No	No	No	No
**Moshaverina A et al[2013]**	No	No	No	No
**Yang H et al[2013]**	No	No	No	No
**Moshaverina A et al [2014]**	Yes	No	No	No
